# Quality evaluation methods of chinese medicine based on scientific supervision: recent research progress and prospects

**DOI:** 10.1186/s13020-023-00836-3

**Published:** 2023-09-30

**Authors:** Zhangmei Chen, Chi Teng Vong, Tiejun Zhang, Chun Yao, Yitao Wang, Hua Luo

**Affiliations:** 1 Macau Centre for Research and Development in Chinese Medicine, State Key Laboratory of Quality Research in Chinese Medicine, Institute of Chinese Medical Sciences, University of Macau, Macao, 999078 People’s Republic of China; 2 Guangxi University of Chinese Medicine, Nanning, 530001 People’s Republic of China; 3 College of Pharmacy, Guangxi Medical University, Nanning, 530021 People’s Republic of China; 4 State Key Laboratory of Drug Delivery Technology and Pharmacokinetics, Tianjin Institute of Pharmaceutical Research Co., Ltd, Tianjin, 300462 People’s Republic of China

**Keywords:** Chinese medicine, Scientific regulation, Quality evaluation methods

## Abstract

Traditional Chinese medicine (TCM) is increasingly getting attention worldwide, as it has played a very satisfactory role in treating COVID-19 during these past 3 years, and the Chinese government highly supports the development of TCM. The therapeutical theory and efficacies of Chinese medicine (CM) involve the safety, effectiveness and quality evaluation of CM, which requires a standard sound system. Constructing a scientific and reasonable CM quality and safety evaluation system, and establishing high-quality standards are the key cores to promote the high-quality development of CM. Through the traditional quality control methods of CM, the progress of the Q-marker research and development system proposed in recent years, this paper integrated the research ideas and methods of CM quality control and identified effective quality parameters. In addition, we also applied these effective quality parameters to create a new and supervision model for the quality control of CM. In conclusion, this review summarizes the methods and standards of quality control research used in recent years, and provides references to the quality control of CM and how researchers conduct quality control experiments.

## Introduction

Traditional Chinese medicine (TCM) refers to a general term for the medicines of various Chinese ethnic groups, which reflects the understanding of life, health and disease of the Chinese. It has been used to maintain health, prevent, diagnose and treat diseases, and boost health. It is widely used for the treatment of diseases and health care. TCM has been gradually accepted by most of the countries in the world. The chemical compositions of Chinese medicine (CM) prescriptions are complex, the raw materials are unstable, so the quality is difficult to control. CM refers to the medicine used to treat diseases under the guidance of the theory of TCM, and it has the functions of rehabilitation and health care. CM mainly includes medicinal plants, animals and minerals. The lack of relevant standardized quality control can result in uneven quality of CM and affect its curative effects and international status, so regulatory science is important for the quality control of CM. The core of drug regulatory science is the innovative practice of new standards, new tools, and new methods for developing, predicting, evaluating, selecting, and verifying drug safety and effectiveness. Regulatory science focuses on policies, systems and legal systems governed by the medical sector and social construction.

This review summarized the quality evaluation techniques and methods involved in CM quality control in recent years, involving the whole process of the production cycle of CM, from the origin, processing, efficacy, metabolomics, and pharmacokinetics of medicinal materials. The above methods are combined with Q-marker to establish safe, effective and scientific TCM theory-related quality evaluation standards and technical methods, and then integrate with mathematical calculation, thus providing new research ideas for quality control. This can ensure good clinical efficacy and safety of CM.

## Quality control mode of CM based on raw materials

### Based on the origin of medicinal materials

The origin of medicinal materials refers to the type of plant from which specific medicinal materials come, such as family, genus, species, and whether it is included in books like “*Flora of China*”. The origin of medicinal materials can be studied by textual criticism, which is summarized through relevant research on medicinal plants. For example, Zhao and colleagues resolved the controversy over the origin of the Qu Aurantii Fructus base source plant from Quzhou City, Zhejiang Province, and determined its Chinese and Latin names, which can be provide reference and futher quality control for the Qu Aurantii Fructus selected for subsequent research [1].

Authentic medicinal materials deriving from long-term clinical practice and summary of TCM, are a kind of high-quality Chinese medicinal materials with significant regional characteristics. They are a unique comprehensive criterion for distinguishing the high-quality Chinese medicinal materials that originated from ancient times. For example, the famous “Four Major Huaiqing Medicines”, with high quality and curative effects, refers to Dihuang (*Rehmannia glutinosa Libosch*), Shanyao (*Dioscorea Opposita Thunb*), Niuxi (*Achyranthes bidentata Bl*), and Juhua (*Chrysanthemum morifolium Ramat*), which are produced in Boai, Mengxian, Qinyang and other places under the jurisdiction of Henan Province, while the high quality of “Eight famous herbal drugs in Zhejiang” is produced in Hangzhou. Yue and colleagues reported that the contents of active ingredients in CM from different origins varied greatly. There are many influencing factors, including climate, temperature, precipitation, acidity and alkalinity of the soil [2]. The establishment of quality standards for authentic medicinal materials and primitives requires consideration of the temperature, humidity, light, soil and other habitat characteristics of the production areas, which can be used as standards to guide the production of authentic medicinal materials based on inorganic elements (e.g. sodium, iron, cadmium, and titanium) [3], organic chemical components [4], and microorganisms, which help to quickly distinguish authentic medicinal materials from non-authentic medicinal materials [5, 6]. A large number of studies have shown that authentic medicinal materials have excellent varieties, large yields and significant curative effects. Therefore, the origin of medicinal materials can be used as a preliminary criterion for controlling the quality of medicinal materials.

### Based on the authenticity of Chinese medicinal slices

**Fig. 1:**
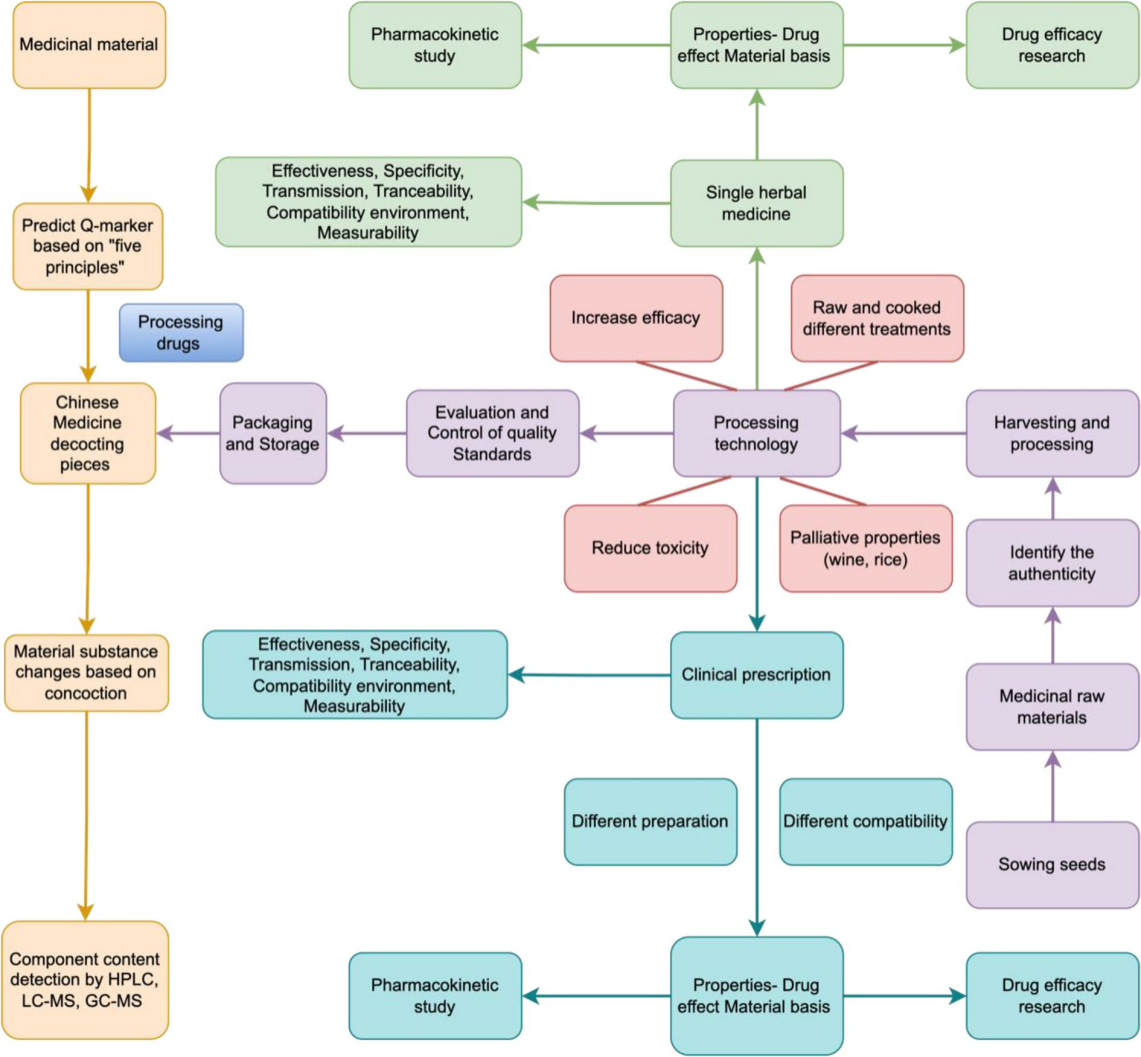
Flowchart of quality control of Chinese medicinal slices. This figure illustrates every link of Chinese medicinal slices, from planting Chinese herbal medicines to processing into decoction pieces, and finally to the clinical applications of different processed products and compatible decoction pieces. Every link is very important for quality control, especially the part that correlates the detection of the active ingredients of CM with drug efficacy

The quality of CM is controlled by multiple processes, such as medicinal material planting, appropriate processing of Chinese medicinal slices, preparation of extracts, and production of CM. During these processes, adulteration, such as non-medicinal parts mixed with medicinal parts and forgery will seriously affect the quality of Chinese medicinal slices. The authenticity of Chinese medicinal slices can be determined by microscopy, physical and chemical identification, chromatography, liquid spectroscopy, and DNA barcoding. Figure 1 briefly describes the planting of CM from seeds, processing, and the use of decoction pieces for clinical practice. We need to strictly control every link in order to produce CM with high quality, and the curative effect of CM can be ensured to be significant. Appropriate improvements to previous research strategies can enable CM quality control to be more time-saving, labor-saving, and money-saving.

### Based on CM processing

The medicinal properties of CM determine its effects in clinical application, but its processing can change the original medicinal properties and efficacy. The efficacy of CM is mainly affected by processing and metabolism, and its efficacy is positively correlated with its quality. Good-quality Chinese medicine has better efficacy, and the CM with mild properties has a shorter metabolism time and is quickly digested and absorbed, which is intuitively displayed. For example, Coptidis rhizoma is cold in nature, but its bitter-cold nature can be suppressed by adding wine [7], ginger juice [8] and Euodia fructus juice [9]. Besides, processing can also reduce the toxicity and side effects of CM, and its analgesic effect is still retained. For example, aconitine, the active ingredient in Aconitum, can be used for analgesia [10]. After processing, aconitine is hydrolyzed into hypoaconitine and aconine, which reduces its toxicity but still retains anti-pyretic and analgesic effects. Moreover, some CMs can also increase their efficacy by processing. For example, when Corydalis rhizoma is processed with vinegar, it can improve its analgesic effects, soothe the liver and relieve depressive effects [11]. Therefore, the processing of CM is a significant guarantee of the safe use of medicine, which is also a necessary means to improve its therapeutic effects. Besides, CM's biological effects, metabolomics and pharmacodynamics after processing could also be considered for its quality control.

## Quality control methods concerning the material basis of CM

At present, a variety of quality control methods have been established to correlate substance bases with drug efficacy. Reversed phase-high performance liquid chromatography (RP-HPLC) is one of the most common determination methods, including HPLC with a diode array detector (HPLC–DAD) [12], HPLC with evaporative light scattering detector (HPLC-ELSD) [13], HPLC [14] with fluorescence detection (HPLC-FLD), HPLC-ultraviolet–visible-spectroscopy (HPLC–UV-Vis) [15]. Based on liquid chromatography [16] combined with other instrumental analyses are also applied to it, including HPLC-mass spectrometry technology (HPLC–MS) [17]. HPLC-high resolution MS and tandem MS (HPLC-HRMS/MS), ultra-high performance liquid chromatography-tandem mass spectrometry (UPLC-MS/MS) [18]. Additionally, other methods are used to qualitatively and quantitatively identify and determine the active ingredients in CM, such as nuclear magnetic resonance (NMR) [19], and gas chromatography-mass spectrometry (GC–MS) [20]. These methods are generally considered simple, effective, innovative, high separation efficiency, high sensitivity, low potential interference, and short run time, thereby contributing to the development of quality control. NMR quantifies the molecular weights of animals and some organic and inorganic components, which are suitable for highly specific recognition, selective distinction of homologues, and detecting counterfeiting and adulteration of drugs [21]. In contrast, the disadvantage of the above quality control methods is too many toxic organic solvents [22]. From the perspective of green analytical chemistry, UV spectrophotometry alone has the advantages of environmental protection, low energy consumption, and less analysis time, which is a safer analysis method [23].

### Quality control method of CM based on its material basis and properties and efficacy

The reason why CMs can target the disease and play therapeutic roles is that each CM has certain characteristics and functions. The ancients called it the bias of CM, which means that the bias of the CM is used to correct the excess or decline of Yin and Yang in the disease, but the ancients failed to conduct in-depth research on the material basis of drug action. At present, the basic principle of drug action can be explained by the partial of CM combined with the material basis, which results in a high-level summary of the therapeutic effects of CM. Among them, the partiality of treating diseases is various, and its complex properties and functions are summarized into four qi, five flavors, ups and downs, nourishing and reducing, meridians, poisonous and non-toxic.

Wang and colleagues used the quantitative and visual analysis method of the literature to mine and summarized the published literature, and analyzed the chemical compositions, pharmacological effects and clinical applications of the 416 flavors of the heart, liver and lung meridian CMs, and analyzed their relationships between three of them. According to the mutual relationship between meridians, it was found that the CMs of each meridian contain flavonoids, terpenoids and volatile oils, which have anti-inflammatory, anti-bacterial, analgesic and other pharmacological effects, and are used clinically to treat fever, inflammation, cold and other diseases [24]. Taking the modern research of CM as the starting point, the relationship between chemical compositions, characteristics and curative effects of CM should be comprehensively considered, so as to reveal the rationality, scientificity and practicality of the theory of CM clinical application, thus returning to the viscera and meridians. However, due to the complex composition and a lot of information on CM, many factors can affect the accuracy of the results from the composition research, so it can be considered to perform relevant research from the direction of the active ingredient-target-disease of the bioactive compounds to evaluate the quality of CM.

### Quality control method of CM based on its material basis and biological effects

Biological activities, such as diffusing the lung, blood-activating, stasis-resolving, anti-bacterial, and anti-inflammatory effects, could be used to evaluate the biological effects of CM. Therefore, existing quantification of index components and fingerprints combined with the biological effect evaluation are used to identify active compounds from CM, which could achieve scientific quality control and assessment of CM. For example, Zhang and colleagues assessed the quality of multiple batches of rhubarbs using the biological potency assay and chemical chromatogram fingerprint with the maximum antagonism rate as a reference for the quality control of wine-processed rhubarb products, finding that different batches have different qualities and biological potencies. Besides, they also identified three active compounds with the highest biological potency [25]. According to the classification of the efficacy of different CMs, the biological activity determination methods should be specifically selected to accurately reflect its clinical therapeutic effects. Therefore, this method can accurately identify the active compounds with corresponding biological efficacy of CM, and these compounds can be used as the standards for the quality control of CM. In addition, this method deserves systematic and in-depth research to establish a scientific evaluation method with apparent use objects, advanced detection methods and strong operability.

### Quality control method of CM based on its material basis, metabolism and metabolomics

Metabolomics is an important part of systems biology, which refers to the qualitative and quantitative analysis of metabolites contained in organisms under specific conditions. It is now widely used to study active compounds in CM. The metabolomics platforms currently used include LC–MS, NMR, GC–MS, UPLC-MS/MS [26], UPLC-triple quadrupole composite linear ion trap mass spectrometry (UPLC-Q-Trap/MS) [27], and ultra-high performance liquid chromatography-quadrupole-electrostatic orbitrap mass spectrometry (UHPLC-Q-Exactive/MS) [28]. Compared with other metabolic platforms, researchers are more willing to choose UHPLC-Q-Exactive/MS to solve the problems of the material basis of drug action and the complex system of CM due to its higher resolution, sensitivity and higher peak reproducibility, and rapid qualitative or relative quantitative analysis of multiple compounds at the same time. Ma and colleagues established a strategy to discover quality control markers by mining the metabolome of toad venom. They identified chromatographic components by LC–MS/MS, while metabolomics was used as well to ensure quality consistency [29]. Moreover, Sericea Burck is a South African medicinal plant whose leaves can be used to treat menorrhagia and its roots for infertility treatment, and can be made into skin care products. Mulaudzi and colleagues used HPTLC chemical fingerprint and metabolomics to analyze its root bark for quality control and intraspecies chemical variation. Although using white light in HPTLC to observe TLC plates has limitations, this shortcoming can be overcome by carefully inspecting images corresponding to the locations of individual samples with known concentrations of labeled compounds [30].

### Quality control method of CM based on network pharmacology, material basis and biological effects

The biological effects refer to the impact of certain external factors, such as chemicals and Chinese herbal medicines, on organisms, which can be obtained through direct observation. It is generally used to evaluate environmental quality, such as organochlorine pesticide residues. Therefore, the biological effects of CM provide a good strategy for quality control. Based on network pharmacology, the impact of the CM on the appearance of the human body can be directly investigated by the effective substances combined with biological effects. For example, skin suppuration was caused by bruises can reduce swelling and generate new skin by externally using CM wine. Wang obtained visual data by network pharmacology to evaluate the possible pathways or potential targets of the Mingshi formula for preventing and treating low-grade myopia in children, and recorded the adverse reactions and safety indicators (That is biological effects) during the research process, and then identified the active ingredients from Mingshi formula [31]. This method can shorten the time for the whole process of CM quality control with the assistance of network pharmacology, which can quickly find the effective material basis corresponding to its biological effects.

### Quality control method of CM based on its material basis, properties and active chemical components

Currently, there are many studies investigating the relationship between the properties and components of CM, which can be used as the research direction of CM quality control in the future. The nature of CM can be understood as Chinese medicinal, physical and chemical properties. However, it still needs to clarify the diversification of toxic substances after CM compatibility. It is an excellent choice to apply the association of material base, nature of medicines and components to CM quality control after processing, for example, studying the content of chemical components of Shengdihuang (Rehmannia glutinosa) before and after the processing. The results showed that the levels of total polysaccharides, catalpol, rehmanniaside A and rehmanniaside B were decreased after processing raw rehmannia glutinosa, which has changed the nature of raw rehmannia from clearing heat and cooling the blood to nourishing yin and tonifying blood of cooked rehmannia glutinosa [32, 33].

## Research progress on the quality control methods of CM based on Q-markers

### The overall quality control method of CM related to Q-marker

Clinical formulations have properties, such as uniqueness, combination environment, efficacy, and measurability. Therefore, the focus of quality control is based on the research of the medicinal properties-efficacy-material basis between different processed products and different combinations. The compositions of CM can be first identified by HPLC fingerprint, and then chemical consistency between different batches of CM is evaluated as a quality control marker. More importantly, the corresponding components representing the efficacy of CM can be found through fingerprint-efficacy relationship analysis, which can be used as markers to further control the effective consistency between different batches of CM [33]. The content mentioned above is about the five principles for selecting Q-markers. Therefore, when researchers choose the Q-maker of CM as a quality control indicator, they can comprehensively consider the above reasons. In order to enable readers to understand the research related to CM and Q-marker, we summarized the detection instruments and strategies used for Q-marker research involving CM in the past 10 years in Table 1.

**Table 1:** Examples of studies on Q-markers of medicinal materials and decocting pieces or compound prescriptions

Drug/Prescription name	Processed/composition	Quality marker	Detection equipment	Strategy	Refs
Periplocae Cortex	The dried root bark of Periploca sepium Bge	4-methoxybenzaldehyde-2-O-β-D-xylopyranosyl-(1 → 6)-β-D-glucopyranoside; Periplocin; Periplocoside S-4a; Periplocoside H1 etc	UPLC/Q-TOF MS; UPLC-TQ-MS	Plant metabolomics and network pharmacology	[34]
Centipeda minima	Dried whole herb	Arnicolide D; Brevilin A	HPLC	Chemometric analysis; Network pharmacology and molecular docking simulations	[35]
Typhae Pollen	Carbonized pollen	Isorhamnetin-3-O-Neohesperidoside; Isorhamnetin-3-O-Rutinoside; Kaempferol-3-O-Neohesperidoside; Naringenin; Quercetin; Isorhamnetin	UPLC-QTOF-MS; UPLC-TQ-MS	Chemometric method; systems pharmacology and two high-throughput zebrafish models: cerebral hemorrhage model and thrombus model	[36]
Panax notoginseng	Shatter the roots	Notoginsenoside R1, Ginsenoside Rg1, Re, Rb1 and Rd	HPLC	Integrating chemical profile with pharmacological activities	[37]
Rhubarb	Rice wine	Rhein	UPLC-HRMS	Pharmacokinetic studies; efficacy experiments; Non-targeted and targeted data mining	[38]
Burdock	Stir-fried ripe fruit	Artiin	LC–MS; UFLC-MS/MS	Combining the traditional and modern medicinal effects with the chemical composition	[39]
Ziziphi Spinosae Semen	Frying mature seeds	Spinosin; 6′″-feruloylspinosin; Betulinic	UPLC; UPLC-Q-TOF–MS; LC–MS/MS	The qualitative-quantitative in vitro efficacy-molecular docking method	[40]
Corni Fructus	The fruit of the plant Cornus officinalis Sieb. et Zuc	Loganin	HPLC; HPLC-DVD	Through in vivo and in vitro pharmacological experiments	[41]
Cydonia oblonga Mill	Mature fruit of genus Rosaceae	Caffeic acid; Chlorogenic acid; Ellagic acid; Vanillic acid	UHPLC/Q-TOF–MS/MS	Network pharmacology methods	[42]
Glycyrrhiza uralensis Fisch	Dry roots and rhizome	Liquiritigenin; Liquiritin; Isoliquiritin; Isoliquiritiyenin; Licochalcone A; Glycyrrhetinic acid; Apigenin	HPLC–DAD; HPLC-Q-Exactive /MS	A HPLC based on metabolomics for simultaneous quantitative analysis	[43]
Salvianolic acids for injection	Salvianolic acids for injection	Salvianolic acid B; Rosmarinic acid; Lithospermic acid Salvianolic acid D;	UPLC-Q-TOF-M; LC–MS/MS	The target cell extracts and pharmacokinetic studies	[44]
Sparganii Rhizoma	Dried tubers	Protocatechuic acid; P-coumaric acid; Ferulic acid	HPLC–UV	Anti-thrombotic effect studies	[45]
Radix Wikstroemia Indica	Sweat soaking method (Sprayed synthetic perspiration and Dried in the oven)	Daphnoretin; Emodin; Triumbelletin; Dibutyl phthalate; Methyl Paraben; YH-10 + OH	GC–MS; UPLC-Q-TOF/MS	Study of pharmacodynamics; Toxicity and material basis	[46]
Codonopsis radix	Dryer drying; sun drying; shade drying	Tryptophan; Syringin; Cordifolioidyne A; Tangshenoside I; Codonopyrrolidium A; Creoside IV; Lobetyolin;	UHPLC-Diode Array Detector	Comprehensive chemical analysis; pharmacodynamics	[47]
Scutellaria barbata	Whole plant	Scutellarin; Scutellarein; Luteolin; Lpigenin; Hispidulin	UPLC/IM-QTOF-MS; UPLC-UV	Network pharmacology	[48]
Panax ginseng	Processing and steaming	Protopanaxatriol type ginsenoside (Re, Rf, Rg1, 20(S)-Rh1, Rh4, and Rg6); Protopanaxadiol type ginsenosides (Rb1, Rb2, Rb3, Rc, Rd, 20(R)-Rg3, 20(S)-Rg3, and Rk1); Oleanolic acid type ginsenoside (Ro)	LC–MS/MS; QqQ-MS	Chemical analysis	[49]
Schisandra chinensis	Fruit directly sun-dried or steamed and sun-dried	Schisandrol A; Schisandrin A; Schisandrin C and Gomisin N	HPLC–MS/MS; HPLC-Q-TOF/MS	Target metabolomics; bioinformatic analysis	[50]
Alisma orientale	Dried tubers	Alisol A; Alisol B; Alisol A 23-acetate; Alisol B 23-acetate; Alisol A 24-acetate	LC–MS n -IT-TOF; HPLC	Network pharmacology; Bioactivity analysis	[51]
Musk	The dry substance that is secreted from mature male musk deer subumbilical sac gland	Muscone; Cis-9-hexadecenal; Antioxidant 2264; Prasterone-3-sulfate; Androstan-17-one; 1,2-Benzenedicarboxylic acid	LC/MS–MS; GC/MS	Using combined metabolomic and chemometric approach	[52]
Flors Chrysanthemi	Flower head	Chlorogenic acid; 3,5-Dicaffeoylquinic acid; 1,5-dicaffeoylquinic acid; Luteoloside; Apigenin-7-*O*-β-d-glucoside; Luteolin-7-*O*-6-Malonylglucoside	NIRS; HPLC/Q-TOF–MS	HPLC/Q-TOF–MS identification and heat map clustering	[53]
Tripterygium glycosides tablet	Triptergium wilfordii Hook	Wilforine	UPLC-MS/MS	Cytotoxicity study; Pharmacodynamics; Pharmacokinetics study	[54]
Yuanhu Zhitong tablets	Angelicaeda huricae radix and Corydalis rhizome	Tetrahydropalmatine; α-Allocryptopine; Protopine; Corydaline; Imperatorin; Isoimperatorin; Byakangelicin	RRLC-MS/MS; LC–MS	Integrating chemical and biosynthetic analyses, drug metabolism, and network pharmacology	[55]
Qiliqiangxin capsule	Astragali radix; Ginseng radix; Aconiti lateralis radix; Salviamiltiorrhiza radix; Semen descurainiae lepidii; Alismatis rhizome; Polygonati odorati rhizome; Cinnamomi ramulus; Carthami flos; Periploca cortex and Citri reticulatae pericarpium	Songorin; Calycosin-7-*O*-*β*-D-Glucopyranoside; Astragaloside; Tanshinone IIA; Ginsenoside Re; Hesperidin and Alisol A	UHPLC-MS/MS; UHPLC-Q/TOF–MS/MS	The multi-dimensional “radar chart” mode	[56]
Danlou tablet	Pueraria lobata (Wild); Salvia miltiorrhiza Bge; Alisma Orientale; Astragalus Membranaceus; Ligusticum chuanxiong Hort; The pericarp of Trichosanthes Kirilowii Maxim; Drynaria fortune; Paeonia lactiflora Pall; Allium macrostemon Bge.; Curcuma longa L	Puerarin; Alisol A; Daidzein; Paeoniflorin; Tanshinone IIA	UHPLC-TQ-MS; UHPLC-Q/TOF–MS	Pharmacokinetic; Network pharmacology; Bioactivity evaluation; Drug metabolism	[57]
Tangzhiqing tablet	Nelumbo Nucifera; Paeonia Lactiflora; Salvia Miltiorrhiza; Morus alba; Crataegus Pinnatifida	Nuciferine; Paeoniflorin	UPLC-Q-TOF/MS; LC–MS/MS; HPLC–UV	Clinical trials (dose-exposure–response in human)	[58]
Buyanghuanwu injection	Danggui; Chuanxiong; Chishao	Astragaloside IV; Paeonflorin; Amygdalin; TetramethylpyrAzine; Ferulic acid	RP-HPLC	Polypharmacokinetic model and its similarity approach	[59]
Sijunzi decoction	Radix Ginseng; Macrocephala; Poria; Licorice	Liquirtin; Formononetin; Malonyl-ginsenoside Rb2; Ginsenoside Ro; Glycyrrhetnic acid; Glycyrrhizic acid; 2-atractylenolide; Dehydrotumulosic acid; Isoglabro-lide	UPLC-Q-TOF; UHPLC-Q-TOF	Metabolomics	[60]
Xuezhiling tablets	Radix polygonimultiflori preparate; Cassia seed; Hawthorn; Rhizoma Alismatis	Aurantio-Obtusin; Rubrofusarin Gentiobioside; Obtusinfolin; THSG; SHJ	UPLC-UV–MS; UPLC-UV	Metabolomic study	[61]
Xuefu Zhuyu decoction	Semen Persicae; Flos Carthami; Radix Paeoniae rubra; Rhizoma Chuanxiong; Radix Achyranthis Bidentatae; Radix Bupleuri; Radix Angelicae Sinensis; Radix Rehmanniae; Fructus Aurantii; Radix Platycodonis; Rhizoma Glycyrrhizae	Naringin; Isoliquiritin; Paeoniflorin; Protocatechuic acid; Neohesperidin; Ferulic acid	HPLC; UPLC-PDA	A strategy based on “spider-web” mode	[62]
Wu-Wei-Wen-Tong Capsule	Scutellariae Radix; Huangqin; Yinyanghuo; Pianjianghuang; Guizhi; Fuling	Coumarin; Cinnamic Alcohol; Baicalin; Cinnamic acid; Cinnamaldehyde; Wogonoside; 2-methoxycinnamaldehyde; Epimedin C; Icariin; Baicalein; Wogonin; Baohuoside I; Anhydroicaritin; Germacrone; Pachymic acid	UHPLC-QTOF/MS; UHPLC-UV	Pharmacokinetic study	[63]
Yinlan capsule	Exocarpium Citri Tomentosae; Folium Ginkgo; Herba Gynostemmatis; propolis	Lupeol; Ginsenoside Rb3	LC–MS-MS; UHPLC-MS–MS	Pharmacokinetics research	[64]
HuangQin decoction	Scutellaria baicalensis Georgi; Glycyrrhiza uralensis Fisch; Paeonia lactiflora Pall; Ziziphus jujuba Mill	Paeoniflorin; Baicalin; Scutellarein; Liquiritigenin; Norwogonin; Baicalein; Glycyrrhizic acid; Wogonin; Oroxylin A	HPLC/UV; LC-IT-TOF/MS	System pharmacology	[65]
Buyanghuanwu decoction	Astragali Radix; Angelicae Sinensis Radix; Paeoniae Radix Rubra; Pheretima; Chuanxiong Rhizoma; Carthami Flos; Persicae Semen	Astragaloside IV; Laetrile; Paeonoflorin; Ferulic acid	HPLC–DAD	Chemical analysis; Calculation of Kinetic Parameters and Mathematical Equations	[66]
Qiliqiangxin capsule	Radix Astragali; Aconite Rot; Ginseng; Salvia; Miltiorrhiza; Semen Lepidii Apetali; Cortex Periplocae Sepii Radicis; Rhizoma Alismatis; CartHamus tinctorious; Polygonatum Odorati; Seasoned Orange Peel and Rumulus Ginnamomi,	Astragaloside; calycosin-7-glucoside; Sinapine; Ginsenoside Rg1	HPLC–MS/MS	Pharmacokinetics and pharmacodynamics	[67]
Tangzhiqing tablets	Nelumbo Nucifera; Paeonia Lactiflora; Salvia miltiorrhiza; Morus alba; Crataegus Pinnatifida	Paeoniflorin and Nuciferine	LC–MS/MS; UPLC	Pharmacokinetic study in humans, in vitro dissolution and permeation studies, and in vitro and in vivo relationships between permeation profiles	[68]
Qingzao Jiufei decoction	Mori Folium; Gypsum Fibrosum; Ophiopogonis Radix; Eriobotryae Folium; Ginseng Radix Et Rhizoma; Sesami Semen Nigrum; Asini Corii Colla; Armeniacae Semen Amarum; Glycyrrhizae Radix Et Rhizoma	Chlorogenic acid; Methylophiopogonanone A; Methylophiopogonanone B; Sesamin, Ursolic acid; Amygdalin; Liquiritin Apioside; Liquiritigenin; Isoliquiritin	UHPLC-ESI-Q/TOF–MS	Network pharmacology-metabolomics- pharmacokinetic (PK)/ pharmacodynamic (PD) modeling	[69]
Chaiqin chengqi decoction	Da Huang; Mang Xiao; Hou Po; Huang Qin; Yin Chen; Zhizi	Synephrine; Geniposide; Hesperidin; Sennoside A; Wogonoside; Physcion; Aloe; Sinensetin; Rhein; Honokiol; Magnolol; Emodin	UHPLC; LC–MS/MS; UHPLC-Q Orbitrap/MS; UHPLC-QqQ/MS	Network pharmacology, combining in vivo (AP murine model) and in vitro (pancreatic acinar cell) experiments	[70]

### Quality control method of CM based on component groups with equivalent efficacy

The model of equivalent component groups for CM quality control is based on studying the contribution of “part” to the “whole” activity as a group of active ingredients of CM preparation. And discovering a group of ingredients can basically represent the overall efficacy of the original recipe from a large number of components of CM, which is used as a quality control index. This method improves the existing standards from the perspective of the overall function and correlation with the efficacy of CM to ensure the safety and effectiveness, stability and controllability of CM, thereby providing new ideas and methodologies for CM quality standards. However, there are some disadvantages to using equivalent component groups as the CM quality control model. It requires an effective efficacy model and evaluation, and enrichment of active ingredients. And the formulation of standards needs to be carefully considered regarding the enforceability and differences of herbs, and the content of individual components and their mutual ratios or multiple major components [71].

### The application of cell membrane chromatography (CMC) in quality control of CM

CMC is performed by preparing living cell membranes into cell membrane stationary phases or by culturing cells on specific carrier surfaces to prepare further cell membrane stationary phases, which maintains the integrity of cell membranes so that it can ensure the biological activity of CM. Current cell models for CMC include hepatocytes, vascular smooth muscle cells, epidermal growth factor, cardiomyocytes, and platelet cells [72]. CMC is a bionic chromatography that selectively recognizes active ingredients from CM based on the receptors of the cell membrane by specific binding. It has dual functions of recognition (cell membrane activity) and separation (chromatographic separation), so it is widely used to screen the active ingredients of CM compounds [73, 74]. However, as the membrane receptors attached to the silica gel can easily fall off or lose their activity, the life of the CMC column is short and repeatability is poor. Due to the uncertainty of the number of membrane receptors on the CMC stationary phase, this can affect the accuracy of the analysis results, so cell membrane chromatography is mostly used in conjunction with multi-dimensional chromatographic techniques, such as LC–MS [75], GC–MS, HPLC–MS, and UPLC-MS/MS [76] as a common method for screening, analyzing and identifying target components of specific receptor constituent groups. Lin and colleagues successfully isolated the anti-allergic components in Houpo (*Magnolia officinalis Rehd.et Wils.*) by combining CMC with liquid phase and mass spectrometry [77]. Liu and colleagues established a combined system of mouse Mas-related G-protein coupled receptor member X2 (MRGPRX2)/CMC and liquid chromatography-ion trap-time-of-flight (HPLC-ESI-IT-TOF), which proved to be effective in screening and identifying TCM components that bind to MRGPRX2. In this study, perillae folium was shown to be effective in anti-pseudo-allergic reaction by inhibiting MRGPRX2, a new receptor related to pseudo allergic reaction. Using this established system, they successfully obtained the binding affinities of rosmarinic acid and apigenin to MRGPRX2 (KD value) [78].

### The application of serum medicinal chemistry in quality control of CM

Most drugs are needed to be absorbed into the blood. After being absorbed, distributed and metabolized, they are transported to their targets as prototype components or metabolites to exert their medicinal effects. Some active ingredients of CM are only a part of its components. The active substances and their prototype compounds can really clarify and express the pharmacodynamic substance basis of CM. Serum medicinal chemistry mainly studies the chemical substances in the serum, which can simulate the in vivo process of drugs and avoid possible erroneous conclusions from in vitro experiments. The applicable components of serum medicinal chemistry are invalid during in vitro experiments, but they can be transformed into active ingredients in vivo. The research on serum medicinal chemistry provides technical support for identifying the pharmacodynamic material basis of CMs and improving the quality control standards of CMs [79]. The key to quality control is whether the markers selected as quality control markers are absorbed in vivo. Chen and colleagues showed that after Huangqi Keli treatment in rats, pharmacologically active substances, such as Astragaloside IV, Formononetin, and Calycosin, were detected in the serum [80]. Li and colleagues analyzed the quality markers of Yuquan capsules based on CM serum pharmacochemistry, and used UPLC-Q-TOF–MS and UNIFI systems to detect the active components and metabolites of Yuquan capsules after being absorbed into the blood. 12 prototype components, including Formononetin and Schisandrin A, and 12 metabolized components, such as Ginsenoside and Liquiritin, were identified. 24 ingredients have been shown to exert anti-diabetic activities, which can be used as quality markers of Yuquan capsules, thereby laying the foundation for establishing its quality control system [81]. However, the research method of serum medicinal chemistry has its shortcomings. Firstly, it is not suitable for stimulating the gastrointestinal tract and other drugs that do not pass through the blood. On the other hand, in the process of serum preparation and inactivation, it may lead to the loss and degradation of the active ingredients of CM.

### The application of metabolomics in quality control of CM

In the last 5 years, countries, such as Malaysia [82], Vietnam [83], and the United States, have combined metabolomics with quality control for food or pharmaceutical products. Moreover, the combination of NMR [84], HPLC and metabolomics can effectively control the quality of CM. Suzuki and colleagues showed that NMR-based metabolomics for the quality control of saponins is effective and feasible, so this method can be used to identify the anti-bacterial active components from other natural medicines as well [85]. Metabolomics employs a global analysis approach to comprehensively analyze altered metabolites, thus providing insights into the global state of the entire metabolic process, which fits well with the integrity and systemic characteristics of CM. In another study, rats were given medicinal and food-based citrus herbs, such as Qinpi and Chenpi, by the oral gavage route, and serum was collected after 60 min and used for subsequent qualitative and quantitative examinations. 32 compounds were quantified and used as markers of CM in this study [86].

### The application of multivariate network regression in quality control of CM

At present, multiple network regression is widely used. The quality assessment of fundus image perception based on multiple regression can be used to diagnose various eye diseases [87]. The results showed that the predicted values of biochemical and chemical oxygen demands calculated by using multiple linear regression and other models for water quality parameters were consistent with the actual measured values [88]. Therefore, this method provided a new research idea for the quality control of CM. The parameters of the relevant ingredient contents and the image of the standard medicinal materials can be set through the multiple linear regression model, and the grades of medicinal materials can be divided into good, general and poor qualities to preliminarily predict the quality of CM. Whether the corresponding index component contents have met the requirements, follow-up experimental verification should be conducted.

### Quality control method on toxic substances basis as Q-marker

The safety of medication has always attracted attention from the public, especially the medicinal toxicity of CM. The main reasons for the toxicity of CM are excessive dosage, improper processing, improper compatibility, such as the use of Aconitum [89] and Pinelliae Rhizoma [90]. However, the toxic components of CM can treat some intractable diseases [91]. The toxic ingredient could be the active component, for example, strychnine, the Q-marker of *Strychnos nux-vomica L.*, which is poisonous but effective, and widely used in the clinical treatment of various tumors. Its dosage is controversial among researchers and doctors. If misused, it will threaten the safety of humans. The toxic CM research, in which efficacy and toxicity co-exist, can become a branch independently. Making a systematic safety evaluation, toxic compounds regarded as Q-markers of toxic CM, that is, the toxic compounds basis combined with acute toxicity, long-term toxicity, reproductive toxicity, and genotoxicity and other safety evaluation should be considered [92].

### Pharmacokinetic quality control method of CM related to Q-marker

In the past decade, pharmacokinetics has been one of the hot research topics in quality control. Researchers have found the Q-maker provides a reference for the dosage and interval of CM by pharmacokinetics. Many quality controls of CM are based on pharmacokinetics [93] combined with UHPLC-Q-Orbitrap HRMS [94] or UHPLC-MS/MS [95]. The distribution, metabolism and excretion of CMs’ chemical components in vivo can be further determined by ultra-fast liquid chromatography-mass spectrometry/mass spectrometry (UFLC-MS/MS) [96–103]. The average oral bioavailability reflects the proportion of the administered drug entering the human circulation, which is a good evaluation index. Chen and colleagues showed that an extract of Callerya nitida var. hirsutissima was administered to rats, then blood samples were collected from the retroorbital venous plexus at 0.083, 0.5, 1.0, 1.5, 3.0, 4.0, 6.0, 8.0, 12.0 and 24.0 h for subsequent pharmacokinetic experiments. The values of area under the drug-time curve (AUC) and terminal elimination half-life (T1/2) in this experiment reflected that the chemical components were well absorbed and distributed in the body after oral administration [104]. Therefore, pharmacodynamics is one of the critical components for the quality control of CM, which lays a theoretical foundation for Q-markers and can be widely used in discovering Q-markers for CM to improve the quality control standards and clinical efficacy of CM.

### Quality control method based on mathematical calculations

Mathematical calculations are commonly used in the quality control of CM, mainly including standard deviation (SD), coefficient of variation (CV), and standard deviation index (SDI) [105]. Research has shown that verifying mathematical calculations can avoid errors in reporting, which is widely used in blood routine laboratories. Among them, some studies have established mathematical models based on Fick rules and Noyes-Whitney [44] equations to study the potential Q-markers of CM, which are in line with the “five principles” of Q-markers, namely effectiveness, Specificity, transmission traceability, compatibility environment, and measurability. As an important tool, mathematical statistics is used for data collection, processing and analysis, and conclusion judgement of a large number of detection values. The correct application of mathematical statistical methods is important for scientific management and quality control of products. Huang used mathematical statistical methods to conduct sampling inspections on incoming materials from liquor companies, and controlled the quality of packaging materials during the production process to prevent packaging materials that did not meet the quality standards from flowing into the production line, thus ensuring the quality of product [106]. Applying the mathematical statistical methods in quality management has enhanced the concept of prevention, from post-inspection to pre-control, and it achieved the purpose of quality control and quality improvement. Its shortcoming is that the inference of the whole by partial observation can only reflect the whole to a certain extent, but cannot be exactly the same as the whole, and estimation cannot be guaranteed to be correct. This principle and method can also be used for random sampling inspection of qualified CM. Therefore, it is necessary to develop a new standard based on the mathematical model for the CMs’ quality process from raw materials to product manufacturing, which can greatly promote the development of quality control of CMs’ pharmaceuticals and their preparations.

## Research progress of quality control methods on CM

### Determination of multi-index components

Multi-index component determination is to use a series of representative chemical components to evaluate the whole, or multiple chemical components of a medicinal material to evaluate the pros and cons of the CM. For example, the content of these representative chemical components in a medicinal material is relatively high, or they jointly exert certain biological activities that can represent the medicinal material’s characteristics. When detecting its chemical composition, multiple chemical components can be detected. For example, Wang and colleagues analyzed the multi-index components of Lycii Cortex based on content determination and chemometric methods, and simultaneously determined 8 chemical components from Lycii Cortex, including Kukoamine A, Kukoamine B, and Chlorogenic acid. This method can distinguish Lycii Cortex samples from different origins. In addition, Du and colleagues have established an in vitro dissolution method for Rhodiola Granules to determine multi-indicator components in Rhodiola Granules. Therefore, the detection of multi-index components can better reflect the interaction of different components and difference in dissolution. This method is simple, accurate and reliable, which provides a methodological reference for the quality evaluation of Rhodiola Granules and other CM [107]. Luo and colleagues established a method for determining the dissolution degree of Liuwei Dihuang concentrated pills by using the content of multiple chemical components as indicators. The results showed differences in the dissolution rate of different pharmaceutical factories. This method provides a scientific basis for improving the quality evaluation of Liuwei Dihuang concentrated pills [108]. Wu and colleagues measured the content of multiple index components in Wenjing Decoction and analyzed their quantitative transfer process, which provided a basis for further research on the complex chemical components of Wenjing Decoction, and provided a quality control basis for the subsequent development of its related preparations[109]. Overall, the multi-indicator content determination method is simple and easy with good accuracy, stability and repeatability, and can provide a reference for the quality standards of medicinal materials. The disadvantage is that some medicinal materials from some origins have overlapping chemical components and their content difference is not very obvious, which cannot be well distinguished. Therefore, the content of inorganic elements and infrared spectra can be further studied to distinguish between them.

### Chromatographic fingerprint recognition

#### Chromatographic fingerprint identification of CM

Chromatographic fingerprint refers to CM, cell DNA and protein treatment with certain analytical means to obtain the chromatogram or spectrum, which is mainly divided into three categories. CM chromatographic fingerprint is one of the categories. The establishment of chromatographic fingerprint provides a better reference for CMs’ quality control and evaluation. Huang and colleagues studied the fingerprints of Puerariae Lobatae Radix from different origins and attributed them to 6 active ingredients, which provides a more comprehensive reference for the quality control and evaluation of Puerariae Lobatae Radix [110]. Wang and colleagues studied the chromatographic fingerprint of Sinopodophyllum hexandrum with HPLC, founding 20 peaks and identifying 12 compounds. This method can be used for quality control and provides the scientific basis for the clinical application of CM; it also provides a reference for studying the differences in efficacy of two different parts of a CM [111]. Wu and colleagues conducted chromatographic fingerprint studies on Glycyrrhiza uralensis in various combinations of Mahuang decoction. They found that the peak area of some common peaks in different matching fingerprints showed a corresponding decrease or increase, indicating that the content of a specific chemical substance in CM was somewhat affected after matching. This method is stable and reproducible, providing a reference standard for the quality control of Mahuang decoction [112]. Zhu and colleagues established the fingerprints of 24 batches of mulberry-sourced medicinal materials, such as Mori Ramulus, Mori Folium, Muri Cortex, and Mori Fructus, and analyzed the differences in the fingerprints, summarized the analysis of “homologous effects” of component differences. It provided a more comprehensive analysis method for quality marking [113].

#### Multivariate and multidimensional quantitative chromatographic fingerprint

Multivariate refers to three or more wholes, and multidimensional refers to the number of squares that are independent and orthogonal to each other in geometric space, which are usually larger than three dimensions. The multivariate multidimensional fingerprint is a technique that uses different analysis techniques but the same separation methods to obtain a stereoscopic multidimensional spatial fingerprint of CM under different detection conditions or different detection principles [114]. Sun and colleagues used HPLC fingerprints and UV fingerprints to monitor the distribution of unsaturated bond chemical components, and used infrared fingerprints and combustion enthalpy to monitor the composition analysis of saturated compounds, thus establishing a multivariate and multidimensional cross-assessment method for the quality of Fangfeng Tongsheng pills [115, 116]. Multivariate and multidimensional fingerprint technology has complementary advantages in principle, and it can reasonably cross-integrate qualitative and quantitative information. It has the technical basis for comprehensive identification of the quality of Chinese patent medicine. The advantage of multidimensional chromatography is that it has higher resolution and peak capacity. Meanwhile, it also has the properties of reality, effectiveness, reliability and operability, which are suitable for separating very complex mixtures [117, 118]. Therefore, it is applied for the separation and identification of CM compounds, especially those CM prescriptions with more than a dozen flavors of Chinese medicinal, and it is amicable to find reference materials. However, there are still some limitations, and it is still impossible to find all the chemical components, especially those with low contents.

#### Spectrum-effect relationship of CM

The spectrum-effect relationship is a scientific method based on the fingerprint of CM to study the correlation between CMs’ fingerprints and activity. This method confirms activity-related peaks and the attribution of the peaks. Its advantage is that it can clarify the material basis of pharmacodynamics to provide directions and ideas for the evaluation method of the intrinsic quality of CM [119–121].

Therefore, in recent years, many studies used the spectrum-effect relationship to evaluate the quality of CM. Ding and colleagues identified eight major chemical components of Lonicera japonica honeysuckle using a combined spectroscopic-effects method, and seven chemical components, including neochlorogenic acid (5-caffeoylquinic acid), chlorogenic acid, 4-dicaffeoylquinic acid, and three isomers of isochlorogenic acid were determined by enzyme-linked immunosorbent assay. All of them have anti-pyretic and anti-endotoxic activities [122].

### CM with “concept of great quality”

The research on the quality standards of CM based on the idea of “concept of great quality” is a complex systematic project. Xiao pointed out that the “concept of great quality” is a preliminary research idea that needs to be constantly revised and improved [123]. There are various quality control modes, which combine sensory, biological and chemical methods for evaluating the quality of CM. In terms of content detection, the direct correlation between content and drug efficacy was discussed based on the spectrum-effect relationship. The research on the dose–effect relationship covers the principle of “sovereign, minister, assistant and courier” in the compatibility of CM Formula. That is, the sovereign medicinal usually has the most dosage and provides the principal curative action on the central syndrome. The minister medicinal takes the second place to help strengthen the principal curative action. Assistant medicinal relieves secondary symptoms through the action of the sovereign ingredient. Courier medicinal directs action to the affected meridian. The amount of Chinese medicinal has a difference in treating diseases, which might come from different varieties, different product specifications and grades, different particle sizes of decoction pieces, different index component content, dosage and weighing medicinal materials, and pharmacodynamic component content. Therefore, no matter in clinical or scientific research, it should be considered that the quality and dosage of medicinal materials are unified. And for basic research, for example, quality control of CM is finally reflected in the results of clinical trial verification. It is an indispensable part of further guiding clinical and rational drug use.

### Double standard evaluation

**Fig. 2:**
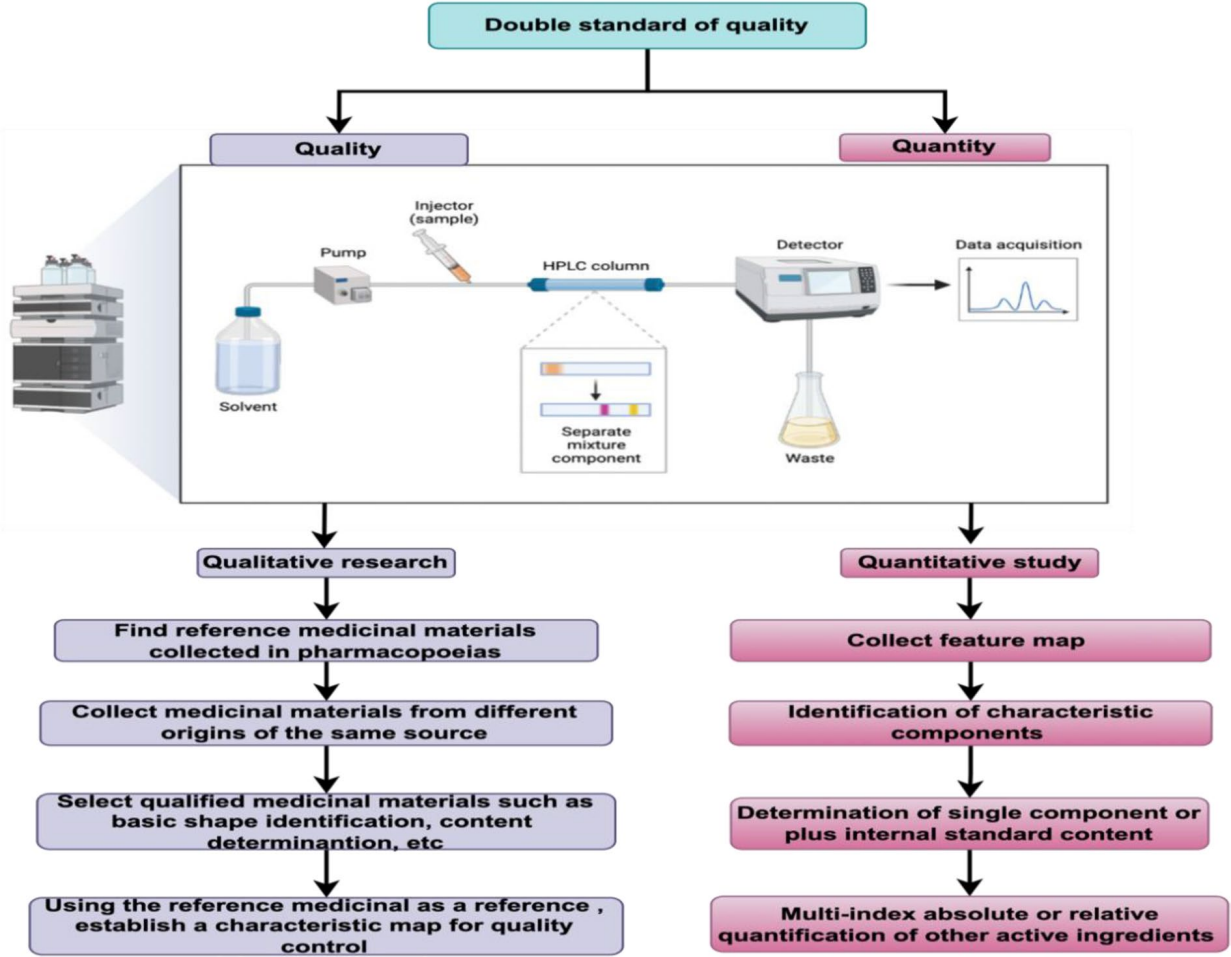
The strategy for the double standard of CMs’ quality control. This flow chart represents the key steps of CMs’ quality control with double standards. Both qualitative and quantitative research include sample preparation, sample loading, detection, data processing and other processes. The difference lies in qualitative research, which mainly determines the composition of substances, that is, the content contained in a certain CM. For CMs’ chemical composition, quantitative research aims to find the most representative compound in large-scale research

Double standard evaluation includes two aspects of CMs' qualitative and quantitative research. The separation techniques used for qualitative and quantitative analysis of CM include ultraviolet detection, fluorescence detection, evaporative light scattering detection, mass spectrometry and nuclear magnetic resonance. Among them, the qualitative research is to collect multiple batches of medicinal materials with the same base source and different origins through the standard medicinal materials included in the pharmacopoeia, and perform base source shape identification, thin layer chromatography identification, reverse high performance liquid phase and other content determination of these medicinal materials to establish quality control standards for the characteristic map of the medicinal materials. Quantitative research is the identification of the characteristic peak components in the characteristic map, meaning the determination of other active components with a single component or the content of the internal standards, and a quality control standard for the absolute or relative quantification of multiple indicators (Fig. 2). Wang and colleagues used a double-standard evaluation method to establish a fingerprint of CM to evaluate the antioxidant components of Citri Reticulatae Pericarpium, the method used in this article is also applicable to the analysis of other CM, which provides a comprehensive and practical method for the quality control of CM [124]. Due to the combination of qualitative and quantitative characterization strategies, the double-standard evaluation system provides an effective and reliable model for complex CM systems. And it is used to establish quality standards for single medicinal materials [125] and CM prescriptions, such as Gualou Xiebai Banxia Decoction [126], Zuo Guiwan [127], You Guiwan [128], and Bao Yuan decoction [129].

## Current challenges and future perspectives

The existing research is focused on the key quality attribute parameters of Q-marker for the whole process of the CM industry; it is not only used to guide the selection of medicinal materials, optimization of process parameters, quality control and drug efficacy evaluation, but also to establish a quality evaluation technical system of CM compounds based on the complete expression of quality attributes through Q-markers or Bio-markers, which is of great significance to ensure the quality of CM and promote the development of the CM industry. However, there are still some technical challenges in establishing a scientific system that conforms to the characteristics of CM: (1) There are still many scientific questions about how to embody the theory of CM compatibility, which have not yet been explained. How does the “sovereign, minister, assistant and courier” rule embody their weight-efficacy and make them consistent? (2) How do we strengthen the correlation between quality control indicators and functions, and whether the chemical compositions can accurately correspond to a certain biological effect? (3) How to characterize scientifically and display comprehensively its quality attributes for a breakthrough of developing simple and easy detection methods to make the quality of Chinese medicinal materials safer and more effective? (4) The measurability of some complex drugs or ingredients, such as veterinary drugs, mineral drugs, polysaccharides and other macromolecular substances, still has some technical challenges. For some CMs without reference substances, it is the most challenging problem at present. How do we find the characteristic fragment ions of the Chinese medicinal material ions, oxides and hydrolysates? (5) Big data information and processing technologies related to the quality of CMs are still being developed and improved, including data statistics and analysis in quality management, and whether the original data uploaded by researchers is real and how to monitor them. Although the instruments in most laboratories are equipped with the conditions to be connected to the network, the data of such laboratories cannot be changed, but for a small number of laboratories that do not have a network connection, relevant researchers can be arranged to follow up regularly, for example, the experimental data are generated, copied and saved as soon as possible, and review them later.

## Conclusion

In conclusion, the quality control of Chinese medicinal materials should be guided by the theory of TCM, which constructs a pharmacological model that conforms to clinical functions and indications. It also carries out dose–effect evaluations corresponding to curative effects, and reveals the core modules and key target pathway systems of drug regulation through multi-group integrated analysis of target organs or targets, thereby providing a scientific theoretical basis for interpreting their biological mechanism of action and clinical value. With the help of liquid quality identification technology and molecular network analysis, researchers should systematically analyze the chemical components, in vivo exposure components and their metabolites to sort CM, thus laying the material foundation for the traceability and delivery of Q-markers. The quality control standards should be focused on the core mechanism or effect module, through network pharmacology prediction of in vivo exposure components, correlate the analysis based on activity and components, and spectral efficiency screening based on effect pathways, identification of Q-markers based on affinity mass spectrometry, which links Q-markers or Bio-markers targets to drug efficacy. Researchers should integrate the above research findings of systems biology and chemomics, and select key Q-markers to design and synthesize small molecule probes. Moreover, researchers should also develop a drug tracing and target capture research system based on chemical biology, deeply study the interaction between active ingredients and targets, and interpret the key scientific concept of TCM treatment from “protein, cell, animal and clinical” levels. It is also recommended to select key quality attribute parameters that can reflect clinical advantages and product characteristics, classify the properties of CM and chemical components in CM, and select appropriate quality control methods according to the various research methods mentioned above. For example, for CM containing volatile chemical components, GC/GC–MS is used for the quality control of the CM. Furthermore, controllable and evaluable research should be performed on the whole process of “medicinal materials, processing, preparation, and in vivo study” to standardize product quality, and highlight the values of CM varieties. Therefore, this review provides a reference research paradigm for the scientific study and application of quality attributes of complex systems.

## Data Availability

Not applicable.

## Abbreviations

TCM: Traditional Chinese medicine
CM: Chinese medicine
Q-marker: Quality marker
HPLC: High performance liquid chromatography
HPLC–UV-Vis: HPLC-ultraviolet–visible spectroscopy
UPLC-MS/MS: Ultra-high performance liquid chromatography-tandem mass spectrometry
GC–MS: Gas chromatography-mass spectrometry
UPLC-Q-Trap/MS: Ultra-high performance liquid chromatography-triple quadrupole composite linear ion trap mass spectrometry
HPLC–MS: High performance liquid chromatography-mass spectrometry technology
HPLC–DAD: HPLC with a diode array detector
HPLC-ELSD: HPLC with evaporative light scattering detector
RP-HPLC: Reversed-phase HPLC
HPLC-HRMS/MS: HPLC-high resolution MS and tandem MS
NMR: Nuclear magnetic resonance
LC–MS: Liquid chromatography spectrometry
UHPLC-Q-Exactive/MS: Ultra-high performance liquid chromatography-quadrupole-electrostatic orbitrap mass spectrometry
HPTLC: High-performance thin-layer chromatography
TLC: Thin-layer chromatography
MRGPRX2: Mouse Mas-related G-protein coupled receptor member X2
HPLC-ESI-IT-TOF: Liquid chromatography-ion trap-time-of-flight
SD: Standard deviation
CV: Coefficient of variation
SDI: Standard deviation index
